# The Role of Young Child Formula in Ensuring a Balanced Diet in Young Children (1–3 Years Old)

**DOI:** 10.3390/nu11092213

**Published:** 2019-09-13

**Authors:** Jean-Pierre Chouraqui, Dominique Turck, Gabriel Tavoularis, Constance Ferry, Christophe Dupont

**Affiliations:** 1 Paediatric Nutrition and Gastroenterology, Division of Pediatrics, Woman, Mother and Child Department, Centre Hospitalier Universitaire Vaudois (CHUV), 21 rue du Bugnon, 1011 Lausanne, Switzerland; 2 Division of Gastroenterology, Hepatology and Nutrition, Department of Paediatrics, Lille University, 59037 Lille, France; Dominique.turck@chru-lille.fr; 3 Jeanne de Flandre Children’s Hospital and Faculty of Medicine, University of Lille, INSERM U995, 59037 Lille, France; 4 CREDOC (Centre de Recherche pour l’Etude et l’Observation des Conditions de Vie), 142 rue du Chevaleret, 75013 Paris, France; tavoularis@credoc.fr; 5 36 rue des Cormiers, 77690 Montigny sur Loing, France; constanceferry@hotmail.com; 6 Pediatric Gastroenterology Department, Necker Enfants-Malades Hospital, Paris Descartes University, 75015 Paris, France; christophe.dupont@aphp.fr

**Keywords:** cow’s milk, DHA and ARA intake, early childhood, diet quality, iron intake, nutrient intake, nutritional adequacy, protein intake, sodium intake, young child, young child formula

## Abstract

During the nutritional vulnerable period of 1–3 years of age, nutrient intake is often inadequate due to an unbalanced diet. Young child formula (YCF) has been proposed as a means of improving nutrition in this age group. We compared the food consumption and nutrient intake of 241 YCF consumers (YCF-C) to those of 206 non-consumers (YCF-NC), selected from among the children enrolled in the Nutri-Bébé survey, an observational cross-sectional survey, conducted from 3 January to 21 April 2013. Food consumption and nutrient intake were analyzed from a three-day dietary record. The YCF-C < 2 years group had a protein (−8 g/d; *p* < 0.0001) and sodium (−18%; *p* = 0.0003) intake that was lower than that of YCF-NC, but still above the respective EFSA (European Food Safety Authority) Average Requirement (AR) or Adequate Intake (AI). At all ages, the YCF-C group had higher intakes of essential fatty acids (*p* < 0.0001), vitamins C (*p* < 0.0001), A, D, and E (*p* < 0.0001), all B vitamins (*p* < 0.001) except B12, iron (9 vs. 5 mg/d, *p* < 0.0001), reaching the Dietary Reference Values (DRVs, AR or AI), but similar DHA and ARA intakes. Getting closer to the reference values proposed by EFSA required at least 360 mL/d of YCF. The consumption of YCF may help infants and children at risk of nutrient deficiencies to meet their nutritional requirements. However, protein, sodium, and vitamin A intakes remained above the EFSA DRVs, and DHA, ARA, and vitamin D remained below.

## 1. Introduction

Early childhood (1–3 years of age) is a period of rapid growth and development, with a gain of approximately 25% in height and 50% in weight occurring during this period [1]. While milk remains a major food, this is a transition period from weaning foods towards a family diet, rendering children vulnerable to nutrient inadequacy [2,3,4]. Taking into account the different studies reported so far, the European Food Safety Authority (EFSA) considered, in 2013, that dietary intakes of alpha-linolenic acid (ALA), docosahexaenoic acid (DHA), iron, vitamin D, and iodine (in some European countries) are low in infants and young children living in Europe, when compared to the dietary reference values (DRVs) [5]. EFSA, therefore, proposed to pay particular attention to ensuring an appropriate supply of ALA, DHA, iron, vitamin D, and iodine in infants and young children with inadequate, or at risk of inadequate, status of these nutrients [5]. More recently, the French Nutri-Bébé survey, covering 1035 infants (<1 year) and young children (1–3 years) who were not breast fed at the time of the survey, revealed a high intake of protein, sodium, and vitamin A, and a low intake of fat, ALA, DHA, iron, vitamin D, and vitamin E [3,4]. These inadequacies were attributed to the early weaning and/or abandonment of milk formula, as well as to the consumption of an unbalanced diet with excessive use of semi-skimmed cow’s milk and meals intended for adults [3,4].

Young Child Formula (YCF, so called “Growing-Up Milk” (GUM)), an alternative to cow’s milk (CM) or breast milk for children 1–3 years of age, are marketed as products specifically formulated for the nutritional needs of young children aged 1–3 years [6]. They are fortified with several nutrients, including iron, vitamin D, and essential fatty acids (EFA), and they contain less protein, saturated fat, and sodium than CM. YCFs have been widely available in many countries since the early 90s. Previously classified as foods intended for a particular nutritional use (so-called “dietetic foods”), since July 2016 they have been considered common foods, fortified with certain nutrients and targeting a specific sub-group of the population (young children) [6]. The European Society for Paediatric Gastroenterology, Hepatology, and Nutrition (ESPGHAN) Committee on Nutrition considered, after reviewing the literature, that there was limited data on the nutrient intakes of young children consuming YCF, and the potential role of YCF in their diets [7]. Therefore, in a recent position paper, the Committee considered it of interest to determine whether YCF intake could correct (and to what extent) some of these deficits, as compared to CM. A few months later, the results of a study conducted in New Zealand and Australia asserted that the consumption of YCF was associated with increased likelihood of meeting nutrient requirements [8]. Owing to the small number of studies conducted to date, it made sense to assess the nutritional intake of YCF-consuming children in Europe, using the data from the Nutri-Bébé study. 

Among the 1035 non breast-fed children enrolled in the Nutri-Bébé study, YCF was consumed by 52.4% at the age of 12–17 months, 54.3% at 18–23 months, 30% at 24–29 months, and 27% at 30–35 months [2]. Mean daily consumption decreased with age from 233 g at 12–17 months to 68 g at 30–35 months. The main objective of the present study was to describe the food consumption and nutrient intake of children aged 1 to 3 years living in France, and their association with the consumption of YCF. The secondary objective was to investigate the minimal daily intake of YCF necessary to obtain the best benefits with respect to the adequacy of nutrient intake. 

## 2. Methods

The analyses were performed using data from the Nutri-Bébé 2013 study, a cross-sectional food consumption survey. The design, recruitment procedure, methodology, and global results of this study have been reported in detail elsewhere [2]. According to French law, no ethical approval was required for this study as it was conducted by an official polling institute, TNS Sofres, in full agreement with the guidelines laid down in the Declaration of Helsinki and the French data protection act, and additionally the current research did not involve invasive procedures or therapeutic interventions. The study was registered on ClinicalTrials.gov (NCT03327415).

### 2.1. Study Sample 

The survey was conducted from 3 January to 21 April 2013, by TNS Sofres, an official polling institute in full agreement with the guidelines laid down in the Declaration of Helsinki and the French data protection act. According to the French legislation ethical approval for this study was not required since the current research did not involve invasive procedures or therapeutic intervention. The study has been registered on ClinicalTrials.gov (NCT03327415). 

**Figure 1:**
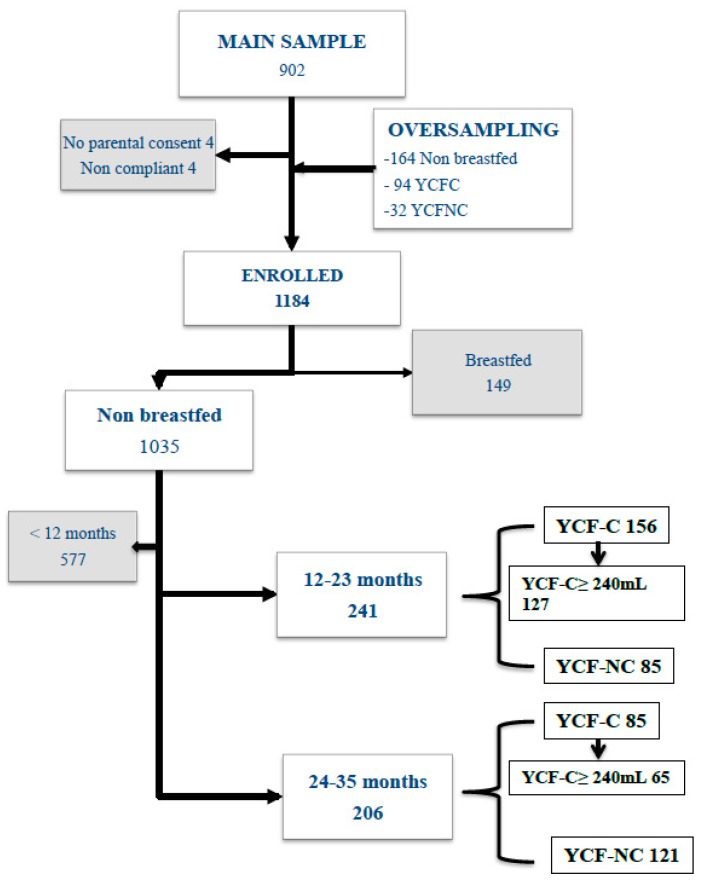
Flowchart of the study sampling to select children consuming young child formula (YCF-C) vs. children who did not consume any formula (YCF-NC).

In brief, a randomly selected sample of 1184 healthy children was enrolled (Figure 1). From the 1035 non-breastfed children, 998 were included in the intake analysis [2], with 241 aged 12–23 months and 206 aged 24–35 months, selected for the current case-control study. The inclusion criteria for selection of children in the initial sample were that they were at least 1 year old, and either consumed YCF or did not consume any infant formula or YCF at all. According to the design of the study the only exclusion criteria was some breastfeeding (*n* = 19/1184). First, intakes were compared between YCF consumers (YCF-C) who were consuming at least 1 mL of YCF daily and non-consumers (YCF-NC), who consumed neither YCF nor infant formula, but rather plain cow’s milk or dairy products. Second, the YCF-C aged 12–23 months were separated into three groups with a daily consumption levels above 240 mL (i.e., the volume of one bottle generally proposed to a child of this age): 240–360 mL, 360–480 mL, and >480 mL, with the aim of determining the cut-off value for adequate intake. Due to the sample size, the children aged 24–35 months were split only into two groups with daily consumption levels of 240–360 mL and >360 mL. Children consuming less than 240 mL were not taken into account in this second step of the study. 

### 2.2. Assessment of Dietary Intake 

After giving informed consent, the parents of the children in the sample were given a diary, a measuring jug (Curver 1 L; gencod 3253920404008), a photographic tool kit, kitchen measuring tools, and scales to assess their child food consumption [2]. The TNS Sofres investigator, namely a dietitian, explained to them the use of these different tools. Subsequently the parents were asked to describe in the diary the nature and the amount of food that their child consumed over a non-consecutive three-day period, including one weekend day. These recordings over three days took place in between two face-to-face interviews; the first to explain the procedure, and the second to review the diary, check for completeness, and clarify details. During the second visit, the child was weighed (naked with a clean diaper or underwear). 

### 2.3. Data Analysis 

Food and drink intake were analyzed by the Centre de Recherche pour l’Étude et l’Observation des Conditions de Vie (CREDOC) (Research Centre for the Study and Observation of Living Conditions) [2]. 

The foods consumed were described and grouped in food categories, according to the level of consumption. In addition to formula, YCF, milk and cheese (hard and semi-hard cheese, spreadable cheese, molten cheese), and other milk products were distinguished between smooth dairy products (yogurt, fresh cheese, Swiss type cream cheese) and milky desserts (cream, custard, mousse, pudding etc.). Apart from potatoes, starchy foods included bread, pasta, semolina, rice, bulgur, quinoa, beans, peas, and lentils. In addition to ham, other delicatessen foods included pâté and sausages. The industrial ready meals included all the canned or frozen prepared dishes that can be used such as pizza, pies, “quiche”, lasagna, ratatouille, meat- or fish-based dishes, etc. Finally, all foods that were rarely consumed, with a mean intake lower than 15 g/d, were grouped under the heading “miscellaneous”. This included eggs, fish, added fat, ketchup, mayonnaise, and confectionary. The intake of all nutrients with an available content is listed in the various tables depicted in the Results section. We could not investigate the intake of certain trace elements, such as iodine and selenium. Special attention was given to nutrients revealed to be above or below the DRVs in previous global analyses, including protein, sodium, vitamin A, fat, ALA, DHA, iron, vitamin D, and vitamin E [3,4]. The nutrient content of consumed foods and drinks were obtained from the French CIQUAL food composition table [9]. For homemade mixed food dishes, calculation of nutrient intakes was based on the recipe reported by the parents and the final amount consumed. For “foods intended specifically for babies” (FSB), as well as for industrial ready meals, the nutritional composition was obtained either from the manufacturers or from product labelling, or by calculation based on the average composition of the ingredients. The mean nutritional compositions of the YCF that were consumed by study participants are shown in Table 1 (61 different brands). The mean food consumption of the two age group’s values were calculated as follows:

$$A_{a}=\frac{\sum_{k\in a}(\frac{\left(A_{k,1}+\;A_{k,2}+A_{k,3}\right)}{3}}{N_{a}}$$

where *A_a_* is the intake of food in each age group, Ak,1, Ak,2, and Ak,3 are the contributions of each individual on the 1st, 2nd, and 3rd day of the survey, and *N_a_* is the size of the age group. 

The nutrient intakes were calculated as previously described [3,4], depending on the formula or milk consumed and compared to the DRVs set by the EFSA either as Average Requirement (AR) or as Adequate Intake (AI) [5,10,11,12,13,14,15,16,17,18,19,20,21]. The contribution of YCF to the nutrient intakes was calculated for each one by reporting the mean intake of this nutrient provided by the YCF relative to the mean total intake of that nutrient.

### 2.4. Statistical Analysis 

The results are expressed as the mean daily intake (DI) ± standard deviation (SD) of each age group. The statistical analysis was performed using the SAS 9.2^®^ software (http://support.sas.com/documentation/installcenter/922/, Cary, NC, USA). The analysis of the quantitative data was based on ANOVAs and the Student’s *t*-test. Differences were considered significant at *p* level < 0.05. The relationship between the daily consumption of YCF and the daily intake of a nutrient was established using the Pearson correlation coefficient. 

## 3. Results

### 3.1. Characteristics of the Population and Food Consumption 

**Figure 2:**
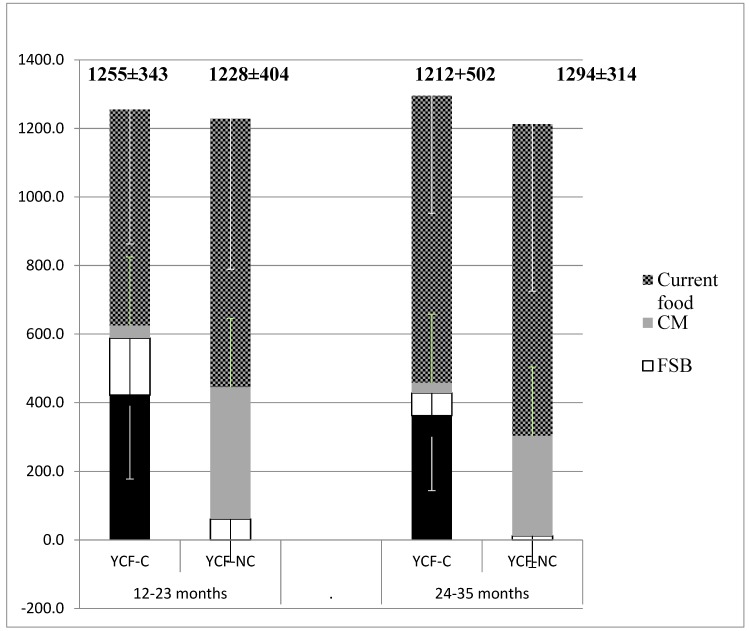
Mean (± SD) daily intake (g) of YCF, foods intended specifically for babies (FSB), CM and common foods in YCF-C (*n* = 241) and YCF-NC (*n* = 206) according to age group.

They were 271 children in the YCF-C group and 206 in the YCF-NC group (Figure 1). Table 2 gives the mean weight of the children and the volume of YCF or CM consumed according to age. No difference was found in weight between the YCF-C and YCF-NC groups. Consumption of YCF was higher than that of CM, but the difference was only significant from 2 to 3 years of age. The mean total intake of food (liquid and solid) in each age group was similar in both YCF-C and YCF-NC groups, whatever the age group (Figure 2). The proportion of feeding allocated to solid foods in the YCF-C group was smaller in the two age groups (respectively 63% vs. 69%, *p* = 0.006, and 70% vs. 76%, *p* < 0.001). The share of “foods intended specifically for babies” (FSB) was higher in the YCF-C groups at all ages (respectively 166 ± 196 vs. 61 ± 126 g/d, and 66 ± 126 vs. 12 ± 92 g/d) (*p* < 0.001). Table 3 provides a detailed description of these differences. Together, YCF and FSB accounted for 47% vs. 5% of the total daily food intake in the 12–23 months group and for 33% vs. 1% in the 24–35 months group. In the YCF-C groups, FSB alone accounted respectively for 35% and 28% of the daily food intake. Among common foods, apart from CM, the main differences were that the YCF-C group aged 12–23 months less often consumed delicatessen foods (4.4 times less), potatoes (1.4 times less), starchy food and pulses (1.4 times less) industrial ready meals (1.9 times less), cookies and cakes (1.5 times less), fruit juice/soda (7 times less), instant chocolate powder (6 times less), drinking water (1.5 times less), and added salt (3 times less) while they consumed at least twice as many vegetables. Those in the YCF-C group over 2 years old consumed less industrial ready meals (1.3 times less), fruit juices (2 times less), chocolate powder (2 times less) breakfast cereals (3 times less), and drinking water (1.3 times less), while they consumed 1.3 times more vegetables. The total daily intake of liquid foods, including drinking water, was similar in both groups (12–23 months: 643 mL vs. 616 mL; 24–35 months: 638 mL vs. 646 mL).

### 3.2. Comparison of Nutrient Intake between YCF-C and YCF-NC 

The mean DI and the corresponding DRVs are depicted in Table 4. 

The mean total energy intake (TEI) was on average 8% higher in the YCF-Cs over age 2-years group, and not before this age. The protein DI was lower (–8 g/d) in the 12–23 months YCF-C group and tended to be similar in both groups after 2 years (–3 g/d), always remaining more than three times the EFSA Average Requirement (AR) [5]. None of the children had a protein intake lower than the recommendation. While mean fat intake was similar in both age groups, that of linoleic acid (LA) and α-linolenic acid (ALA) was significantly higher in the YCF-C group; +77% and +71% in the 12–23 months group and +75% and +82% in the 23–35 months group. A total of 21% of YCF-C aged 12–23 months and 10% of those aged 24–35 months had an LA DI that reached the EFSA average intake (AI) vs. 5% and none of the YCF-NC group (*p* < 0.0001) [5]. ALA AIs were reached by 37% and 23% of the YCF-C group vs. 9% and 1% of the YCF-NC group (*p* < 0.0001). Long chain polyunsaturated fatty acid (LC-PUFA) intake was similar in both groups. The DI of vitamins B, C, A, D, and E, and of iron and zinc, was higher in the YCF-C groups. The vitamin A DI was far above the EFSA AR [15], because of high intakes of both carotene and retinol. The iron DI was nearly three times higher in the YCF-C groups, and reached the EFSA AR only in these groups [13]. Sodium intake was 18% lower in the younger YCF-C group before 2 years of age but not after that age, and remained more than three times higher than the EFSA AI [5]. The YCF-C group consumed 50% more fiber than the YCF-NC group before the age of 1 year and more than the double afterwards; intake nearly reached the EFSA AI [5]. The YCF-C of the two age groups were more likely to reach the EFSA DRVs for Vitamin B1, B2, B3, B9, B12, A, E, iron, and zinc (Table 5).

### 3.3. Contribution of YCF Consumption to Intake of the Most Outstanding Nutrients (Figure 3)

Depending on the age of the child and volume of YCF consumed daily, YCF consumption accounts for approximately the following intakes: more than 80% of dietary vitamin D, 40% to 70% of EFA and vitamin E, 40% to 65% of iron, 25% to 50% of fat, 30% to 50% of sugars (i.e., mono and disaccharides), 20% to 40% of total energy, carbohydrates and vitamin A, 14% to 30% of protein, less than 30% of DHA, and 7% to 20% of sodium.

### 3.4. Relationship between YCF Consumption and Nutrient Intake (Table 6) 

A positive correlation was observed in both age groups between YCF consumption and intake for total fat, LA and ALA, sugars and iron, while a negative correlation was observed for sodium. 

### 3.5. Determination of the Minimal Beneficial Intake of YCF (Table 7 and Table 8) 

All groups had a similar mean energy intake except for the YCF-C group aged 24–35 months, who had a higher intake when consuming more than 360 mL. Only YCF-Cs under 2 years had a mean protein intake that was significantly lower than that of the YCF-NC group. On average, more than 80% of the children had carbohydrate intakes exceeding the recommendations, including the lower YCF consumption group. YCF-Cs aged more than 2 years consuming more than 360 mL/d had the highest intake. No difference was seen in the total mean fat intakes, which as a percentage of TEI, were always below the AI. In the two age groups, only 16% and 28% of the biggest YCF consumers reached the AI. Mean daily intake of EFA increased with the intake of YCF and was higher than that of YCF-NCs in both age groups. The mean daily intake of LA and ALA reached EFSA AIs only in YCF-Cs consuming more than 480 mL before 1 year, and fell short of the EFSA AIs in YCF-C aged more than 2 years consuming more than 360 mL. Among children <2 years consuming more than 480 mL of YCF daily, 38% reached the AI for LA and 57% for ALA. Respectively, 28% and 46% of children ≥2 years and consuming more than 480 mL of YCF daily reached the EFSA AIs for LA and ALA. All YCF-Cs had vitamin mean intakes higher than YCF-NCs, with vitamin A intake well above the EFSA AR for 76% and 89% of the two age groups in the YFC-NC group, and 100% of YCF-Cs. Mean iron and zinc daily intake reached the EFSA ARs in all YCF-Cs. Having 100% of children <2 years with an iron intake equal to or exceeding the EFSA AR required a daily intake of YCF above 480 mL. When the daily intake of YCF is between 360 and 480 mL only 79% achieve this goal. For children older than 2 years, it required an YCF intake above 360 mL/d. Overall, 92% to 100% of the sodium intakes exceeded EFSA DRVs. 

## 4. Discussion

While YCF use is increasing in many countries [6], only three studies have so far evaluated nutrient intake in YCF-Cs compared to YCF-NCs [8,22,23]. The EFSA has concluded, “YCF are one of several means to increase n-3 PUFA, iron, vitamin D and iodine intakes in infants and young children living in Europe with inadequate or at risk of inadequate status of these nutrients. However, other means, such as fortified cow’s milk, fortified cereals and cereal based foods, supplements, or the early introduction of meat and fish into complementary feeding and their continued regular consumption are efficient alternatives to increase intakes of these nutrients. The selection of the appropriate form and vehicle through which these nutrients are provided in the diet will depend on national dietary habits, health authorities, the regulatory context, and the choice of caregivers” [6]. The ESPGHAN Committee on Nutrition considers determining whether YCF intake could correct (and to what extent) some of these deficits as compared to cow’s milk a topic of interest [7]. 

The present study shows that the replacement of CM by YCF contributes, in part, to better fit to the expert recommendations on nutrient intake of young children. It also showed that the two feeding groups (YCF-C and YCF-NC) differed not only in the type of milk or formula consumed, but also in their global feeding practices. Compared to parents of YCF-NCs, parents of YCF-Cs were more likely to offer their child, less solid foods and, at least for those aged 12–23 months, a lesser intake of delicatessen foods, meat, cookies, cakes, fruit juice/soda, instant chocolate powder, and added salt, and a higher intake of vegetables. In addition, YCF-C children consumed more FSB. Altogether, this has resulted in lower intakes of protein and sodium, and higher intakes of LA, ALA, iron, zinc, and vitamins B, C, A, D, and E. It should be noted, moreover, that the two populations (C and NC) are different in other ways. YCF-Cs were different in that they were shown to be more often breastfed in the first months of life, and their mothers were more likely to belong to the highest socio-professional category [2]. Although higher intakes of essential fatty acids, iron, and sodium correlated with YCF consumption, it is not known to what extent these and other aforementioned intakes more in line with the recommendations were due to the healthier dietary patterns and FSB consumption offered to YCF-C children, rather than to the YCF consumption itself. The contribution of YCF consumption to nutrient intake is extremely variable, depending on the quantity consumed but also on the composition of the formula. The YFC consumed in the present study, as well as the dairy products based on YCF, were numerous and varied leading to a significant variability in their composition. However, the results of a recent randomized, controlled study including 83 children aged 1 year in New Zealand and Australia, and using only one brand of YCF, gave similar data in assessments performed from 18 to 23 months of age [8]. In our study, the total quantities of food, liquid, and energy were similar in both groups. The composition of YCF gives rise to a lower intake of proteins, as already reported by others [8,22,23]. Whatever the group, the protein intake remains high, well above the AR, and is not correlated with YCF intake [5]. The potential impact of such levels of protein intake on current or future health is a matter of debate, and has already been discussed regarding the consequences on weight gain and renal solute load [3]. Against all expectations, but like in other studies [8,22,23], both groups had a similar fat intake, while it has been shown that 88% of the CM consumed by YCF-NCs is semi-skimmed [2]. This can be explained by the fact that the YCF-NCs consumed not only more meat, but also more fatty foods of the adult type such as industrial ready meals, delicatessen foods, cookies, and other bakery products. We previously reported that during the second year of life 81% of children could consume the same food as their parents [2]. This increases to 95% after the age of 2 years. 

The most relevant results, in line with previous reports [8,22,23,24], are the higher intakes of EFA and iron, as well as the lower sodium DI, by the YCF-Cs. These intakes were correlated with the intake of YCF. However, few of the YCF-Cs had EFA intakes reaching the EFSA AI, and there was no difference in the LC-PUFA intake. Less than 20% of the YCF used were supplemented with LC-PUFA at the time of the study. The iron intake reached, on average, the EFSA DRVs only in the YCF-C group. In three European countries, the prevalence of iron deficiency (ID) in children who received mainly YCF was 5.4% vs. 19.7% in children receiving mainly CM (*p* < 0.001) [25]. A European longitudinal study showed the positive correlation between iron intake and YCF consumption [26]. A British computer simulation modeling study reported that inadequacy of iron intake decreased from 53.8% to 2.7% when a matched volume of CM was replaced by YCF, and to 1.1% when CM was replaced by 300 mL of YCF [27]. In the New Zealand-Australian study, 24% of children consuming YCF had an iron intake reaching the AR, vs. only 9% of the CM consuming control group [8]. In this study, after one year of YCF consumption, the children had a higher hemoglobin concentration with an adjusted mean difference of 2.8 g/L (*p* = 0.05) and a higher serum ferritin concentration (adjusted mean difference = 17 μg/L; *p* < 0.0001) [28]. The risk of developing ID was lower (OR = 0.32 (0.09–1.09); *p* = 0.068). A randomized controlled trial has previously been undertaken including 225 healthy 12–20 months old children, to determine whether either an increase in meat consumption or iron fortified milk was the most effective for improving iron status [29]. After 20 weeks, serum ferritin significantly increased by 44% in the fortified milk group and was significantly higher than in controls, while it did not change in the meat group. 

A German randomized study assessed an improvement in the vitamin D status of children 2–6 years of age using YCF instead of CM, with a median daily intake of 234 mL [30]. The high vitamin A intake in the YCF-C groups was also emphasized in the British simulation study [27]. The total vitamin A intake includes retinol and β-carotene. YCF adds only retinol with a content that never exceeds the upper limit set by the EFSA [15]. In fact, the EFSA has recommended to decrease the vitamin A content in formula [31]. Dietary β-carotene is considered a safe source of vitamin A because its intestinal conversion to vitamin A decreases as an oral intake of β-carotene increases, and no known adverse health effect has been associated with its consumption from foods [32,33]. Possible concerns about total vitamin A intake does not apply to non-industrialized countries where vitamin A deficiency is a public health issue, vitamin A-rich foods are not widely available, and vitamin A deficiency may affect approximately one-third of children [34]. 

In our study, a daily consumption above 240 mL of YCF significantly reduced the risk of insufficiency in iron and vitamin E but not EFA, unlike previously reported, nor LC-PUFA [22]. To obtain the relevant beneficial intakes, young children between 1 and 3 years of age should drink at least 360 mL/day of YCF. These consumption levels are higher than those proposed in 2012 (250 mL) [22] or in 2015 in the UK (300 mL) [27]. They are in line with those from an international expert group (200–400 mL) [35] and lower than the Belgian consensus-statement (500 mL) [36] or that of an expert panel in 2013 (400–600 mL for children 1 to 6 years old) [37]. 

The strength of the present study comes from the use of data extracted from the Nutri-Bébé survey, whose methodological quality has been acknowledged [2]. The size of the samples selected and the comparability of the populations in terms of age and weight give robustness. The limitations result from the fact that it is an observational study, using proxy-reported dietary data, which could explain the great inter-individual variability of the data. Since the parents did not receive specific recommendations, the data reflect the daily food consumption of the children according to their parents’ choices.

## 5. Conclusions

YCF allows the most frequent nutritional inadequacies observed in several countries to be overcome, especially iron, and vitamin E [5,35]. YCF consumption can also improve the intake of protein, EFA, vitamin D, and sodium. The results of this study could lead us to consider, with caution, the potential consequences of an unbalanced diet associated with low YCF consumption in terms of the risk of overweight, iron deficiency, and hypertension. To address these issues, very different studies would be needed. While nutrient imbalance is common in many countries, complementary feeding regimens differ and are determined by tradition, empirical behaviors, and availability of foods, including the capacity to afford YCF. As a result, no universal instructions can be formulated. The advice of health professionals must be adapted to each family and child, taking into account the quality of their diet. The use of YCF is not a necessity if the diet provided is balanced as recommended, considering the possible nutrient deficits in each country. YCF is an easy means to compensate, at least partially, for the gap between expectations and reality.

## Figures and Tables

**Figure 3:**
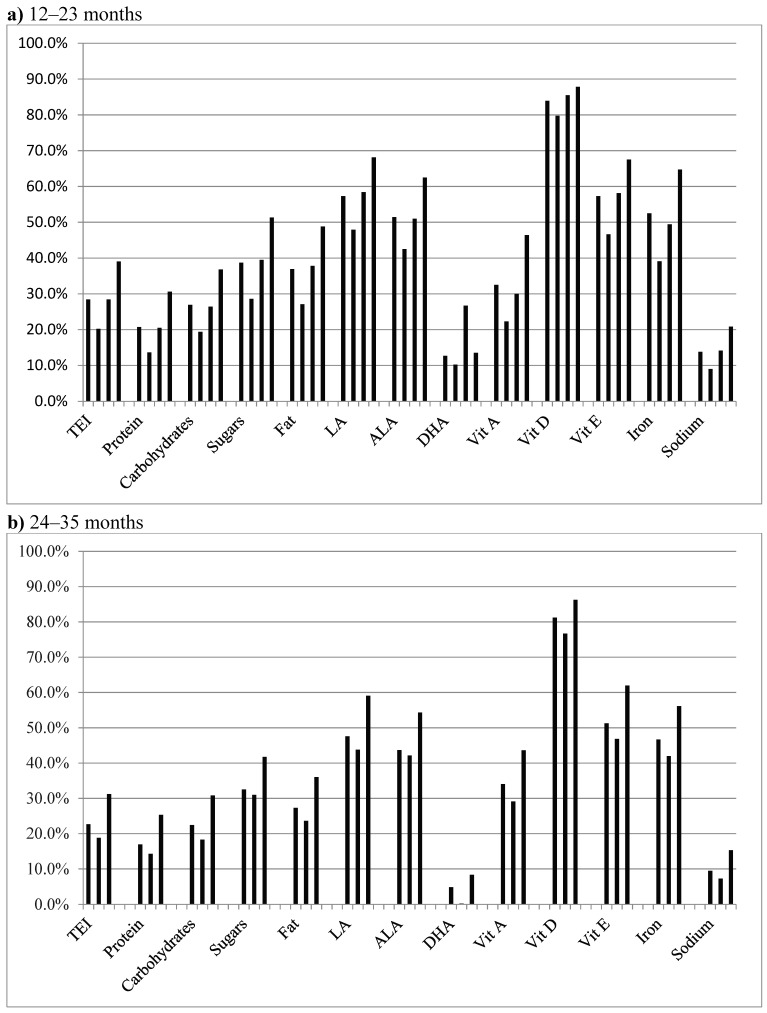
Mean contribution of YCF consumption to energy and outstanding nutrient intakes, according to age group and volume of YCF consumption: (**a**) age group 12–23 months. For each nutrient the vertical bars concern, in order, the total population of the age group, infants consuming 240–360 mL/d, infants consuming 360–480 mL/d, and those consuming 480 mL/d or more; (**b**) age group 24–35 months. For each nutrient the vertical bars concern, in order, the total population of the age group, infants consuming 240–360 mL/d, and those consuming 360 mL/d or more. Sugars: monosaccharides and disaccharides; ALA: α-linolenic acid; DHA: docosahexaenoic acid; LA: linoleic acid; TEI: Total Energy Intake.

**Table 1:** Mean (range) composition of relevant nutrients from cow’s milk (CM) and YCF that were consumed during the survey.

Content per 100 g	YCF (*n* = 61)	Whole CM [9]	Semi-Skimmed CM [9]
Kcal	65.1 (60–74)	64.6	46
Protein (g)	1.8 (1.4–2.7)	3.2	3.3
Carbohydrates (g)	8.1 (7.4–10.1)	4.67	4.83
Fat (g)	2.9 (2.3–3.4)	3.71	1.53
Linoleic acid (mg)	502.9 (283.5–655.1)	57.3	22.2
Alpha-linolenic acid	65.2 (35.1–119.6)	19.1	9.6
DHA	0.7 (0–12) *	0	0
ARA	0.4 (0–8) *	0	0
Calcium (mg)	79.3 (57–132)	112	108
Sodium	29.3 (20–38)	42.3	43.1
Iron (mg)	1.2 (0.7–1.4)	0.05	0.048
Zinc	0.7 (0.5–1.1)	0.38	0.39
Retinol (μg)	66.6 (40–87)	47	20
Vitamin D (μg)	1.3 (0.9–2)	0.03	0.01
Vitamin E (μg)	1.2 (0.5–2)	0.07	0.03

* Only 12 YCF contained DHA and ARA; DHA: docosahexaenoic acid; ARA: arachidonic acid.

**Table 2:** Mean body weight and intake of YCF or cow’s milk (± SD) in each age group.

Age Group	Status		Number	Weight (kg)	Daily Intake (mL) (Median, Range)
12–23 months	YCF Consumers	≥1 mL/d	156	10.9 ± 0.2	422 ± 244 ^a^ (440, 25–1168)
		240–360 mL/d	31	10.9 ± 0.3	294 ± 43.9
		360–480 mL/d	30	10.8 ± 0.3	423 ± 46.5
		>480 mL/d	66	11 ± 0.2	590 ± 155
	YCF Non-Consumers		85	11.2 ± 0.2	384 ± 245 ^a^ (387, 0–917)
24–35 months	YCF Consumers	≥1 mL/d	85	13.2 ± 0.2	362 ± 218 ^b^ (323, 67–1024)
		240–360 mL/d	29	13.1 ± 0.3	284 ± 46
		>360 mL/d	36	13.1 ± 0.3	526 ± 148
	YCF Non-Consumers		121	13.3 ± 0.2	292 ± 275 ^b^ (270, 0–1123)

a: NS; b: *p* = 0.012.

**Table 3:** Food categories consumed by YCF-C and YCF-NC in the two age groups (12–23 months and 24–35 months).

	12–23 Months					24–35 Months				
Food Categories	YCF-C		YCF-NC			YCF-C		YCF-NC		
	(*n* = 156)		(*n* = 85)			(*n* = 85)		(*n* = 121)		
	Mean	SD	Mean	SD	*p*	Mean	SD	Mean	SD	*p*
Total Intakes	1255	342.7	1228	404	0.49	1294.2	313.8	1212	501.7	0.08
Total FSB	588		61.3			427.5		11.9		
Follow on formula	33.7	147.3	0	0	**0.0089**	4.2	61.1	0	0	0.3149
Milk drinks with YCF	32.9	106.7	0	0	**0.0005**	52.9	160.4	0	0	**<0.0001**
YCF	350.2	300.6	0	0	**<0.0001**	304.6	250	0	0	**<0.0001**
Dairy products with YCF	15	42.8	2.2	13.1	**0.0009**	2.2	15.2	0.5	9.6	0.2
Other milky desserts with YCF	8.8	33.2	2.4	14.4	**0.038**	3.4	16.7	0.6	7.1	**0.029**
Baby cereals	9.9	17.8	7.7	20.2	0.3	6.5	14.4	2.3	8.8	**0.007**
Soup mixing YCF and vegetables	16.0	54.2	5.2	36.2	**0.04**	7.6	37.4	0	0	**0.004**
Vegetables or vegetable and cereal mixture	25.7	50	4.4	21.4	**0.0002**	8.8	31.4	2	31.5	**0.045**
Meat-vegetable mixture	37.3	76.9	22.4	65.8	0.06	20.2	54.4	1.7	1.8	**<0.0001**
Fish-vegetable mixture	15.0	42.2	4.8	25.6	**0.012**	5.4	28.6	0.23	4.23	**0.01**
Cooked fruit dessert or compote	31.3	63.4	10.2	34.8	**0.0004**	10	35.2	3.4	32.8	0.07
Other desserts	12.3		1.8			1.6		1.2		
Total Current Food	**666.9**		**1166.7**			**866.7**		**1200.3**		
Cow’s milk	36.9	133.8	384.2	244.6	**<0.0001**	29.7	120.2	291.7	275.4	**<0.0001**
Dairy products	101.7	96.5	106.6	100.6	0.6	100	80.4	100.6	113.4	0.96
Other milky desserts	12	34.3	16.2	54.4	0.35	26.6	50.4	22	49	0.4
Cheese	10	14.9	8.15	16.8	0.3	11.7	15.7	11.3	19.5	0.8
Breakfast cereals	3.4	15.4	5.8	18	0.2	2.5	7.2	7.4	23.45	**0.015**
Biscuits, cookies, crescents, or pastry	17.9	26.4	26.8	30	**0.003**	25.3	29.6	30.6	36	0.15
Vegetables or vegetable and cereal mixture	77.3	124.4	58.4	89.6	0.12	70.8	86	53.2	70	**0.035**
Potatoes	33.2	50.5	46.4	56.4	**0.02**	35.8	48.5	34.5	47.2	0.8
Starchy food and pulses	21	38.4	29.6	42.1	**0.048**	31.7	40.3	37.8	48.45	0.2
Meat and ham	21.5	31.2	30.1	33.7	**0.014**	28.7	30.9	32.4	41.3	0.36
Other delicatessen foods	1.47	5.7	6.6	13	**<0.0001**	6	11.8	8.7	17.2	0.09
Industrial ready meals	14.5	37.4	28.2	56.8	**0.005**	25.9	40.1	40.2	72.4	**0.03**
Fruits	77	66.2	84.9	84.8	0.4	111.4		95		0.15
Fruit or vegetable juices	10.4	34.8	24.7	62.4	**0.0044**	24.1	51.3	50.3	104.8	**0.0056**
Sodas, nectars, syrups, and soft drinks	3.6	19.7	25.3	94.2	**0.0006**	19.4	84.4	38.4	161.7	0.2
Salt	0.13	0.34	0.25	0.45	**0.004**	0.3	0.47	0.28	0.69	0.77
Drinking water	140.7	152.4	208.3	242.3	**0.001**	210.8	203.6	257.1	290.8	0.1
Breakfast chocolate powder	0.66	3.6	4.3	7.6	**<0.0001**	2.4	7.4	5.1	8.9	**0.0028**
Miscellaneous	160.3		156.7			214.9		178.7		

The significant *p* values are in bold.

**Table 4:** Comparison of daily intake between YCF-C and YCF-NC and European Food Safety Authority (EFSA) Dietary Reference Values (DRVs), Average Requirement (AR), or Adequate Intake (AI) in the two age groups.

Nutrients	12–23 Months						24–35 Months					
	DRVs	YCF-C		YCF-NC		*p*	DRVs	YCF-C		YCF-NC		*p*
		Mean	SD	Mean	SD			Mean	SD	Mean	SD	
TEI (kcal/d)	753^a^	974	269	944	296	0.3114	992^a^	1079	297	997	406	**0.0386**
Protein (g/d)	10.5–11.0^a^	35	14	43	14	**<0.0001**	11.5^a^	40	12	43	18	0.0581
Protein (% TEI)		15%	3%	18%	4%			15%	3%	17%	4%	
Carbohydrates (g/d)		132	39	120	41	**0.0044**		142	43	125	55	**0.0015**
Carbohydrates (%TEI)	45%–60%^b^	1	0	1	0		45%–60%^b^	53%	6%	50%	9%	
Sugars (g/d)		73	23	62	22	**<0.0001**		76	24	64	31	**0.0001**
Fat (g/d)		31	11	30	13	0.4145		36	13	34	19	0.1566
Fat (% TEI)	35%–40%^b^	29%	6%	29%	7%		35%–40%^b^	30%	6%	31%	8%	
Linoleic acid (mg/d)		3498	1332	1975	1410	**<0.0001**		3630	1513	2067	1533	**<0.0001**
LA (% TEI)	4%^b^	3.2%	1.2%	1.9%	1.25%		4%^b^	3,0%	0.95%	1,9%	0.99%	
Alpha-linolenic acid (mg/d)		502	204	293	201	**<0.0001**		512	207	281	188	<0.0001
ALA (% TEI)	0.5%^b^	0.46%	0.2%	0.28%	0.19%		0.5%^b^	0.43%	0.14%	0.25%	0.12%	
Ratio LA/ALA	4–10	7	1.9	6.7	3.8		4–10	7.1	2.23	7.4	4.09	
DHA (mg/d)	100^b^	38	67	34	69	0.5913	100^b^	61	107	52	100	0.4142
EPA (mg/d)		27	46	22	47	0.3103		43	81	39	103	0.6551
ARA (mg/d)		22	38	23	27	0.8117		23	25	29	33	0.1031
Fiber (g/d)	10^b^	9	6	8	4	**0.0285**	10^b^	9	4	8	5	**0.0108**
Vitamin B1 (mg/d)	0.5^c^	0.88	0.35	0.7	0.36	**<0.0001**	0.5^c^	0.85	0.26	0.65	0.33	**<0.0001**
Vitamin B2 (mg/d)	0.8^b^	1.42	0.45	1.36	0.47	0.1672	0.8^b^	1.32	0.38	1.21	0.53	**0.0445**
Vitamin B3 (mg/d)	4.6–5.1^d^	9	4.7	7	4.5	**0.0002**	6.2–6.7^d^	9.14	3.74	7.61	5.48	0.0039
Vitamin B6 (mg/d)	0.5^e,^	1	0.5	0.97	0.45	**0.0226**	0.5^e^	1.13	0.40	0.96	0.54	**0.002**
Vitamin B9 (mg/d)	90^f^	199	99	136	77	**<0.0001**	90^f^	194	68	135	84	**<0.0001**
Vitamin B12 (mg/d)	1.5^g^	2.27	1.2	2.14	1.0	0.3168	1.5^g^	2.49	1.10	2.19	1.86	0.0781
Vitamin C (mg/d)	20^b^	97	46	60	49	**<0.0001**	20^b^	98	46	60	59	**<0.0001**
Vitamin A (mg ER/d)	205^h^	853	586	501	412	**<0.0001**	205^h^	704	379	391	403	**<0.0001**
Retinol (mg/d)		419	203	206	137	**<0.0001**		394	223	183	266	**<0.0001**
Beta-carotene (mg/d)		2602	3167	1770	2208	**0.0075**		1859	1902	1249	1872	**0.003**
Vitamin D (mg/d)	15^i^	7.1	3.8	1.21	1.4	**<0.0001**	15^i^	6.40	3.37	1.15	1.46	**<0.0001**
Vitamin E (mg/d)	6^b^	7.4	3.0	3.4	2.2	**<0.0001**	6^b^	7.21	2.59	3.36	2.34	**<0.0001**
Calcium (mg/d)	390^j^	770	205	792	271	0.3946	390^j^	729	191	746	324	0.5675
Phosphorus (mg/d)	250^k^	692	231	825	279	**<0.0001**	250^k^	730	224	796	317	**0.0292**
Magnesium (mg/d)	170^l^	137	56	155	51	**0.0023**	170^l^	149	46	153	54	0.4405
Sodium (mg/d)	170–370^b^	903	477	1109	582	**0.0003**	170–370^b^	1162	525	1189	570	0.6491
Iron (mg/d)	8^m^	9	3.7	5	2.8	**<0.0001**	8^m^	9.06	3.30	5.07	2.89	**<0.0001**
Zinc (mg/d)	3.6 ^n^	6.15	2.0	4.9	1.98	**<0.0001**	3.6^n^	6.24	1.88	4.90	2.29	<0.0001

Sugars: monosaccharides and disaccharides; ALA: α-Linolenic Acid; ARA: Arachidonic Acid; DHA: Docosahexaenoic Acid; EPA: Eicosapentaenoic Acid; LA: Linoleic Acid; TEI: Total Energy Intake; DRVs: ^a^AR [5]; ^b^AI [5]; ^c^AR [17]; ^d^AR [18]; ^e^AR [19]; ^f^AR [20]; ^g^AI [21]; ^h^AR [15]; ^i^AI [16]; ^j^AR [10]; ^k^AI [11]; ^l^AI [12]; ^m^AR [13]; ^n^AR [14]. The significant *p* values are in bold.

**Table 5:** Percentage of YCF-C and YCF-NC whose nutrient intake reached or exceeded the EFSA DRVs in the two age groups.

	12–23 Months			24–35 Months		
Nutrients	YCF-C	YCF-NC	*p*	YCF-C	YCF-NC	*p*
Energy	89%	74%	**<0.0001**	64%	49%	**0.004**
Protein	100%	100%	-	100%	100%	-
Carbohydrates	94%	84%	**0.0008**	94%	83%	**0.0024**
Fat	13%	15%	0.5	16%	20%	0.3
Linoleic acid	21%	5%	**<0.0001**	10%	0%	**<0.0001**
Alpha linolenic acid	38%	9%	**<0.0001**	23%	1%	**<0.0001**
DHA	8%	4%	0.2	16%	17%	0.8
Fiber	28%	19%	**0.04**	38%	20%	**<0.0001**
Vitamin B1	98%	79%	**<0.0001**	96%	71%	**<0.0001**
Vitamin B2	99%	93%	**0.0002**	98%	90%	**0.003**
Vitamin B3	95%	77%	**<0.0001**	84%	53%	**<0.0001**
Vitamin B6	99%	98%	0.09	100%	94%	**0.0073**
Vitamin B9	98%	76%	**<0.0001**	99%	79%	**<0.0001**
Vitamin B12	87%	79%	**0.03**	89%	71%	**<0.0001**
Vitamin A	100%	89%	**<0.0001**	100%	76%	**<0.0001**
Vitamin C	99%	88%	**<0.0001**	100%	89%	**0.0002**
Vitamin D	13%	0%	**<0.0001**	12%	0%	**<0.0001**
Vitamin E	68%	8%	**<0.0001**	74%	6%	**<0.0001**
Phosphorus	100%	100%	-	100%	100%	-
Magnesium	19%	26%	0.06	29%	32%	0.6
Calcium	85%	85%	0.9	78%	74%	0.4
Iron	70%	9%	**<0.0001**	62%	7%	**<0.0001**
Sodium	95%	98%	0.17	99%	100%	0.13
Zinc	98%	79%	**<0.0001**	97%	81%	**<0.0001**

DHA: docosahexaenoic acid. The significant *p* values are in bold.

**Table 6:** Relationship between daily intake of YCF and daily nutrient intake (Pearson correlation coefficient, r).

Nutrient	12–23 Months		24–35 Months	
	r	*p*	r	*p*
Protein	–0.12	0.12	–0.12	0.26
Fat	0.18	0.02	0.28	0.01
LA	0.64	<0.0001	0.50	<0.0001
ALA	0.49	<0.0001	0.42	0.0001
Sugars	0.44	<0.0001	0.43	<0.0001
Iron	0.62	<0.0001	0.76	<0.0001
Sodium	–0.20	0.01	–0.24	0.01

LA: linoleic acid; ALA: alpha-linolenic acid; sugars: monosaccharides and disaccharides.

**Table 7:** Daily nutrient intake of YCF-C compared to YCF-NC aged 12–23 months, according to the intake of formula consumed and to EFSA DRVs, AR, or AI.

Nutrients	12–23 Months											
	DRVs	YCF-C									YCF-NC	
		240–360 mL/d		p C/NC	360–480 mL/d		p C/NC	≥ 480 mL/d		p C/NC		
		*n* = 31			*n* = 30			*n* = 66			*n* = 85	
		Mean	SD		Mean	SD		Mean	SD		Mean	SD
TEI (kcal/d)	753 ^a^	997	297.9	0.28	981	267.1	0.4	985	273.5	0.2	943	296
Protein (g/d)	10.5–11.0^a^	38.2	14.2	**0.042**	34.7	13.6	**0.0008**	34.0	14.7	**<0.0001**	42.98	14.5
Protein(%TEI)		15.3%	3.1%	**<0.0001**	14.0%	3.2%	**<0.0001**	13.7%	3.3%	**<0.0001**	18%	4%
Carbohydrates (g/d)		134.4	40.1	**0.031**	133.6	39.1	**0.044**	133.2	41.2	**0.014**	119.8	40.5
Carbohydrates (%TEI)	45-60%^b^	54.0%	6.2%	**0.008**	54.5%	6.9%	**0.004**	54.0%	6.6%	**0.001**	51%	7.3%
Sugars (g/d)		69.5	19.5	**0.028**	70.3	20.5	**0.02**	80.1	24.4	**<0.0001**	61.6	22.4
Lipids (g/d)		31.2	12.7	0.7	31.6	11.2	0.6	33.2	9.9	**0.07**	30.4	13.0
Lipids (%TEI)	35%–40%^b^	28%	5.6%	0.5	29%	6.1%	0.7	30.6%	5.6%	**0.03**	29%	7.1%
Linoleic acid (mg/d)		3083.2	973.7	**<0.0001**	3450.5	1004.9	**<0.0001**	4124.3	1056.5	**<0.0001**	1974.7	1410.1
LA (%TEI)	4%^b^	2.85%	0.9%	**<0.0001**	3.2%	0.9%	**<0.0001**	3.9%	1%	**<0.0001**	1.9%	1.2%
Alpha-linolenic acid (mg/d)		445.6	130.0	**<0.0001**	512.2	225.3	**<0.0001**	584.4	181.9	**<0.0001**	292.6	200.5
ALA (% TEI)	0.5%^b^	0.4%	0.14%	**<0.0001**	0.5%	0.2%	**<0.0001**	0.55%	0.21%	**<0.0001**	0.28%	0.18%
Ratio LA/ALA	4–10	6.9	1.2	0.8	7.1	2.2	0.9	7.3	1.9	0.6	6.7	3.9
DHA (mg/d)	100^b^	34.0	40.8	0.9	32.8	55.4	0.9	39.4	84.1	0.6	34.0	69.4
EPA (mg/d)		23.4	28.4	0.8	23.3	40.9	0.8	28.9	58.3	0.3	21.9	47.2
ARA (mg/d)		21.3	19.4	0.7	21.5	22.2	0.7	20.6	49.2	0.6	22.8	27.2
Fiber (g/d)	10^b^	10.5	6.6	**0.0002**	10.0	6.5	**0.002**	7.3	4.3	0.6	7.6	3.6
Vitamin B1 (mg/d)	0.5^c^	0.9	0.4	**0.0072**	0.9	0.4	**0.0009**	0.95	0.3	**<0.0001**	0.7	0.3
Vitamin B2 (mg/d)	0.8^b^	1.3	0.4	0.6	1.3	0.4	0.9	1.6	0.5	**0.0007**	1.3	0.5
Vitamin B3 (mg/d)	4.6–5.1^d^	9.0	5.3	**0.019**	9.3	5.1	**0.008**	9.9	4.4	**<0.0001**	7.2	4.5
Vitamin B6 (mg/d)	0.5^e^	1.1	0.5	**0.039**	1.1	0.6	0.2	1.1	0.4	**0.0124**	1.0	0.4
Vitamin B9 (mg/d)	90^f^	219.5	119.2	**<0.0001**	211.4	109.2	**<0.0001**	196.5	67.2	**<0.0001**	136.0	76.9
Vitamin B12 (mg/d)	1.5^g^	2.3	2.0	0.5	2.0	0.8	0.6	2.5	0.9	**0.0124**	2.14	1.0
Vitamin C (mg/d)	20^b^	92.8	45.1	**<0.0001**	102.8	41.2	**<0.0001**	105.9	45.0	**<0.0001**	60.4	49.1
Vitamin A (mg ER/d)	205^h^	873.6	723.6	**<0.0001**	913.9	653.9	**<0.0001**	855.0	441.0	**<0.0001**	501.4	411.6
Retinol (mg/d)		381.9	274.8	**<0.0001**	392.8	133.2	**<0.0001**	502.3	143.8	**<0.0001**	206.4	137.4
Beta-carotene (mg/d)		2950.1	3836.6	**0.01**	3126.8	3429.6	**0.002**	2116.1	2431.8	0.25	1770.2	2207.8
Vitamin D (mg/d)	15 ^i^	5.4	1.2	**<0.0001**	7.0	1.9	**<0.0001**	9.6	3.2	**<0.0001**	1.2	1.4
Vitamin E (mg/d)	6^b^	6.5	1.8	**<0.0001**	7.7	2.9	**<0.0001**	8.8	2.5	**<0.0001**	3.4	2.2
Calcium (mg/d)	390^j^	739.6	232.2	0.2	748.1	194.4	0.3	807.3	183.7	0.6	791.5	270.7
Phosphorus (mg/d)	250^k^	713.6	239.7	**0.01**	648.4	224.6	**0.0002**	696.4	230.9	**0.0002**	824.6	278.9
Magnesium (mg/d)	170^l^	150.3	66.5	0.5	136.5	61.2	**0.041**	132.2	47.0	**0.0005**	155.4	51.2
Sodium (mg/d)	170–370^b^	996.8	402.3	0.2	863.7	364.8	**0.008**	847.3	537.4	**0.0005**	1109.3	581.7
Iron (mg/d)	8^m^	8.9	3.7	**<0.0001**	9.6	2.9	**<0.0001**	10.9	2.9	**<0.0001**	5.0	2.8
Zinc (mg/d)	3.6 ^n^	5.8	1.8	**0.008**	6.1	1.5	**0.0003**	6.9	1.9	**<0.0001**	4.9	1.9

Sugars: monosaccharides and disaccharides. ALA: α-Linolenic Acid; ARA: Arachidonic Acid; DHA: Docosahexaenoic Acid; EPA: Eicosapentaenoic Acid; LA: Linoleic Acid; TEI: Total Energy Intake. DRVs: ^a^AR [5]; ^b^AI [5]; ^c^AR [17]; ^d^AR [18]; ^e^AR [19]; ^f^AR [20]; ^g^AI [21]; ^h^AR [15]; ^i^AI [16]; ^j^AR [10]; ^k^AI [11]; ^l^AI [12]; ^m^AR [13]; ^n^AR [14]. The significant *p* values are in bold.

**Table 8:** Daily nutrient intake of YCF-C compared to YCF-NC aged 24-_36 months, according to the intake of formula consumed and to EFSA DRVs, AR, or AI.

Nutrients	24–35 Months								
	DRVs	YCFC						YCFNC	
		240–360 mL/d		*P C/NC*	≥360 mL/d		*P C/NC*		
		*n* = 29			*n* = 36			*n* = 121	
		Mean	SD		Mean	SD		Mean	SD
TEI (kcal/d)	992^a^	1003.4	277.5	0.9	**1175.6**	**223.2**	**0.0019**	997	406
Protein (g/d)	11.5^a^	38.2	12.0	0.09	**39.4**	**9.2**	**0.15**	**42.9**	**17.6**
Protein (%TEI)		**15.2%**	**2.7%**	**0.0015**	**13.5%**	**2.4%**	**<0.0001**	**17.4%**	**4.5%**
Carbohydrates (g/d)		132.2	35.0	0.4	**155.9**	**35.9**	**<0.0001**	124.7	55.1
Carbohydrates (%TEI)	45%–60%^b^	53.1%	5.6%	**0.027**	**53.0%**	**5.9%**	**0.0211**	**50.1%**	**8.6%**
Sugars (g/d)		68.3	19.2	0.4	**87.5**	**17.6**	**<0.0001**	64.2	31.1
Lipids (g/d)		33.2	13.0	0.8	**41.5**	**10.7**	**0.0041**	33.9	18.5
Lipids (%TEI)	35%–40%^b^	29.3%	5.5%	0.5	**31.8%**	**5.4%**	**0.2168**	30.3%	8.4%
Linoleic acid (mg/d)		3088.7	1357.4	**<0.0001**	**4399.5**	**1229**	**<0.0001**	**2067.3**	**1533.0**
LA (%TEI)	4%^b^	2.8%	0.8%	**<0.0001**	**3.4%**	**1.0%**	**<0.0001**	**1.8%**	**1.0%**
Alpha-linolenic acid (mg/d)		439.8	112.3	**<0.0001**	**604.2**	**210.0**	**<0.0001**	**280.75**	**187.6**
ALA (%TEI)	0.5%^b^	0.4%	0.1%	**<0.0001**	**0.5%**	**0.1%**	**<0.0001**	**0.25%**	**0.1%**
Ratio LA/ALA	4–10	7.02	1.9	0.4	**7.6**	**2.2**	**0.9**	7.5	4.1
DHA (mg/d)	100^b^	56.5	114.5	0.8	**57.6**	**110.0**	**0.7**	51.7	99.9
EPA (mg/d)		40.4	79.2	0.9	**40.6**	**79.9**	**0.9**	38.7	102.9
ARA (mg/d)		22.4	29.6	0.2	**20.3**	**18.6**	**0.07**	28.5	32.7
Fiber (g/d)	10^b^	8.8	3.3	0.2	**8.8**	**4.3**	**0.17**	7.8	4.9
Vitamin B1 (mg/d)	0.5^c^	0.8	0.2	**0.002**	**0.96**	**0.2**	**<0.0001**	**0.6**	**0.3**
Vitamin B2 (mg/d)	0.8^b^	1.2	0.3	0.8	**1.5**	**0.3**	**0.0002**	1.2	0.5
Vitamin B3 (mg/d)	6.2–6.7^d^	8.5	3.2	0.3	**9.6**	**2.4**	**0.0078**	7.6	5.5
Vitamin B6 (mg/d)	0.5^e^	1.0	0.3	0.3	**1.2**	**0.4**	**0.001**	1.0	0.5
Vitamin B9 (mg/d)	90^f^	188.6	72.6	**0.0001**	**206.0**	**59.9**	**<0.0001**	**134.9**	**84.2**
Vitamin B12 (mg/d)	1.5^g^	2.2	1.0	0.9	**2.7**	**0.8**	**0.03**	2.2	1.9
Vitamin C (mg/d)	20^b^	89.3	37.1	**0.015**	**106.6**	**42.1**	**<0.0001**	**59.6**	**58.6**
Vitamin A (mg ER/d)	205^h^	658.6	348.8	**<0.0001**	**822.1**	**344.9**	**<0.0001**	**391.1**	**402.7**
Retinol (mg/d)		318.8	82.3	**0.001**	**512.5**	**221.2**	**<0.0001**	**182.9**	**266.0**
Beta-carotene (mg/d)		2039.2	2116.8	**0.015**	**1857.8**	**1863.2**	**0.03**	**1249.4**	**1871.9**
Vitamin D (mg/d)	15^i^	5.4	1.6	**0.015**	**9.0**	**2.2**	**<0.0001**	**1.1**	**1.4**
Vitamin E (mg/d)	6^b^	6.4	1.6	**0.015**	**8.8**	**2.3**	**<0.0001**	**3.4**	**2.3**
Calcium (mg/d)	390^j^	669.4	201.2	**0.015**	**813.8**	**139.5**	**0.12**	745.9	323.8
Phosphorus (mg/d)	250^k^	683.2	207.5	**0.015**	**741.8**	**168.8**	**0.2**	**796.3**	**316.7**
Magnesium (mg/d)	170^l^	146.6	44.4	**0.015**	**148.5**	**40.8**	**0.5**	**153.2**	**54.1**
Sodium (mg/d)	170–370^b^	1181.4	443.2	**0.015**	**1070.7**	**401.0**	**0.15**	**1189.3**	**569.6**
Iron (mg/d)	8^m^	8.1	2.1	**0.015**	**11.3**	**2.3**	**<0.0001**	**5.1**	**2.9**
Zinc (mg/d)	3.6^n^	5.8	1.7	**0.016**	**7.1**	**1.8**	**<0.0001**	**4.9**	**2.3**

Sugars: monosaccharides and disaccharides. ALA: α-Linolenic Acid; ARA: Arachidonic Acid; DHA: Docosahexaenoic Acid; EPA: Eicosapentaenoic Acid; LA: Linoleic Acid; TEI: Total Energy Intake. DRVs: ^a^AR [5]; ^b^AI [5]; ^c^AR [17]; ^d^AR [18]; ^e^AR [19]; ^f^AR [20]; ^g^AI [21]; ^h^AR [15]; ^i^AI [16]; ^j^AR [10]; ^k^AI [11]; ^l^AI [12]; ^m^AR [13]; ^n^AR [14]. The significant *p* values are in bold.

## Abbreviations

AI	Adequate Intake
ALA	Alpha-Linolenic Acid
AR	Average Requirement
ARA	Arachidonic Acid
CM	Cow’s milk
CREDOC	Centre de Recherche pour l’Étude et l’Observation des Conditions de Vie (Research Center for the Study and Observation of Living Conditions)
DHA	DocosaHexaenoic Acid
DI	Daily Intake
DRVs	Dietary Reference Values
EFA	Essential Fatty Acid
EFSA	European Food Safety Authority
EPA	Eicosapentaenoic Acid
ESPGHAN	European Society for Pediatric Gastroenterology, Hepatology, and Nutrition
FOF	Follow-On-Formula
FSB	Foods Intended Specifically for Babies
ID	Iron Deficiency
IF	Infant Formula
LA	Linoleic Acid
LC-PUFA	Long Chain PolyUnsaturated Fatty Acid
NS	No Significant Difference
RE	Retinol Equivalent
SD	Standard Deviation
SFAE	Secteur Français des Aliments de l’Enfance (French Organisation for Children’s Foods)
TEI	Total Energy Intake
YCF	Young Child Formula
YCF-C	Young Child Formula Consumers
YCF-NC	Young Child Formula Non-Consumers
